# Genetic Trends Estimation in IRRIs Rice Drought Breeding Program and Identification of High Yielding Drought-Tolerant Lines

**DOI:** 10.1186/s12284-022-00559-3

**Published:** 2022-03-05

**Authors:** Apurva Khanna, Mahender Anumalla, Margaret Catolos, Jérôme Bartholomé, Roberto Fritsche-Neto, John Damien Platten, Daniel Joseph Pisano, Alaine Gulles, Ma Teresa Sta. Cruz, Joie Ramos, Gem Faustino, Sankalp Bhosale, Waseem Hussain

**Affiliations:** 1 Rice Breeding Innovation Platform, International Rice Research Institute (IRRI), Los Baños, Laguna Philippines; 2 AGAP Institute, CIRAD, INRA, Montpellier SupAgro, Univ Montpellier, Montpellier, France

**Keywords:** Rice, Drought breeding, Historical data, Genetic trends, Breeding panel

## Abstract

Estimating genetic trends using historical data is an important parameter to check the success of the breeding programs. The estimated genetic trends can act as a guideline to target the appropriate breeding strategies and optimize the breeding program for improved genetic gains. In this study, 17 years of historical data from IRRI’s rice drought breeding program was used to estimate the genetic trends and assess the breeding program's success. We also identified top-performing lines based on grain yield breeding values as an elite panel for implementing future population improvement-based breeding schemes. A two-stage approach of pedigree-based mixed model analysis was used to analyze the data and extract the breeding values and estimate the genetic trends for grain yield under non-stress, drought, and in combined data of non-stress and drought. Lower grain yield values were observed in all the drought trials. Heritability for grain yield estimates ranged between 0.20 and 0.94 under the drought trials and 0.43–0.83 under non-stress trials. Under non-stress conditions, the genetic gain of 0.21% (10.22 kg/ha/year) for genotypes and 0.17% (7.90 kg/ha/year) for checks was observed. The genetic trend under drought conditions exhibited a positive trend with the genetic gain of 0.13% (2.29 kg/ha/year) for genotypes and 0.55% (9.52 kg/ha/year) for checks. For combined analysis showed a genetic gain of 0.27% (8.32 kg/ha/year) for genotypes and 0.60% (13.69 kg/ha/year) for checks was observed. For elite panel selection, 200 promising lines were selected based on higher breeding values for grain yield and prediction accuracy of > 0.40. The breeding values of the 200 genotypes formulating the core panel ranged between 2366.17 and 4622.59 (kg/ha). A positive genetic rate was observed under all the three conditions; however, the rate of increase was lower than the required rate of 1.5% genetic gain. We propose a recurrent selection breeding strategy within the elite population with the integration of modern tools and technologies to boost the genetic gains in IRRI’s drought breeding program. The elite breeding panel identified in this study forms an easily available and highly enriched genetic resource for future recurrent selection programs to boost the genetic gains.

**Supplementary Information:**

The online version contains supplementary material available at 10.1186/s12284-022-00559-3.

## Background

Rice (*Oryza sativa L*.) is one of the world’s major staple crops providing up 20% of the world's dietary energy and feeding more than 3.5 billion people in the world (Wing et al. 2018). Globally, rice is cultivated in an area of 162Mha with an annual production of 755 mt (FAO 2019). Among the area under rice cultivation, more than 30% is under rainfed ecosystems subject to severe drought or water-limited conditions (Dixit et al. 2014). Drought is the major limitation for rice production in rainfed ecosystems leading to yield loss of 13–35% every year (Muthu et al. 2020) and affecting 46Mha of rainfed lowland and 10Mha of upland rice ecosystems in the Asian-Pacific region (Pandey et al. 2007). In Sub-Saharan Africa, drought covers 19% of the total cultivated rice area and is one of the major causes of low rice grain yields (Van Oort and Zwart 2018). Low grain yield under drought conditions is further elevated by the pressing climatic changes due to the increasing frequency of drought severity events, thus further limiting the rice productivity (Lenaerts et al. 2019).

Direct selection for grain yield under drought has been a major focus of the Rainfed Rice Breeding (RRB) program at the International Rice Research Institute (IRRI). Direct selection for grain yield over secondary traits under drought has been proven effective in improving drought tolerance, and as a result, many drought-tolerant rice varieties have been developed (Kumar et al. 2014, 2018; Sandhu and Kumar 2017; Bhandari et al. 2020; Dar et al. 2020). However, due to the complex nature of grain yield under drought; mainly characterized by the small and large effect genes; their epistatic interactions, and interaction with environment; and other abiotic stresses, genetic improvement in drought has been a significant challenge.

Despite these challenges, IRRI has constantly been striving to innovate and develop drought-tolerant rice varieties and disseminate them to farmers in Africa and Asia–Pacific regions. STRASA (Stress Tolerant Rice for Africa and South Asia) Project (2005–2019) at IRRI, funded by the Bill and Melinda Gates Foundation (BMGF) was one of the most successful research programs that led to the successful development and release of more than 30 high-yielding drought-tolerant rice varieties in Asia and Africa (https://strasa.irri.org/). Under this project, imperative efforts were made to incorporate the major drought-tolerant QTLs (*qDTY1.1, qDTY2.1, qDTY2.2, qDTY3.1, qDTY4.1, qDTY12.1 qDTY6.3, *etc*.*) into the background of the mega rice varieties like IR64, Swarna, and TDK-1, which led to the development of several high yielding drought-tolerant rice varieties (Bernier et al. 2007; Venuprasad et al. 2009; Vikram et al. 2011; Mishra et al. 2013; Yadaw et al. 2013; Sandhu et al. 2014, 2019, 2021; Kumar et al. 2014, 2020; Henry et al. 2015, 2019; Dixit et al. 2017, 2020; Sandhu and Kumar 2017; Bhandari et al. 2020; Majumder et al. 2021; Yadav et al. 2021). The most popular drought-tolerant rice varieties include—DRR dhan 42, CR Dhan 801, Sahbhagi dhan in India, Sukha dhan 4, Bahuguni dhan 11 in Nepal, and Katihan 2, Katihan 3, Sahod Ulan 15, Sahod Ulan 19 in the Philippines, Yaenelo 4 in Myanmar, MPTSA and ATETE in Malawi, CAR 14 in Cambodia, and BRRI Dhan66, BRRI Dhan 71 in Bangladesh (https://strasa.irri.org/varietal-releases/drought). Despite these endeavors and the success of the phenotypic selection coupled with marker-based selection strategies, the progress in the genetic improvement of the drought breeding program has been limited. For example, the average estimated rate of genetic gain in rice drought breeding programs in IRRI-India ranges from 0.68% (under non-stress conditions) to 1.8% (severe drought conditions) (Kumar et al. 2021), which is not sufficient to meet future rice demands. Hence, it is crucial to increase rice productivity at a greater rate to ensure food security and prevent potential food crises in the future (Peng et al. 2004; Li et al. 2018). Aiming 1.5% or above genetic gain in rice under drought is a huge challenge and is largely hampered by complex genetics of drought-elevated by extreme climatic changes and increase in the frequency of drought events, and availability of limited land to grow rice.

To suffice the increasing food demands, it is important to breed drought-tolerant rice varieties with expected genetic gains. Rice breeders must be smart to implement the advanced tools and technologies into the existing breeding pipeline and re-design it for accelerating genetic gains. The Accelerated Genetic Gain in Rice Alliance (AGGRi) project funded by BMGF is one of the IRRI’s ongoing projects aimed at modernizing the IRRI-NARES (National Agriculture Research Extension System) rice breeding program and accelerate the genetic gain from the current level of less than 1% to at least 1.5% or above annually.

Genetic gain is an important parameter to check the progress and success of the breeding programs. The genetic gain estimations can be associated with the ongoing breeding program to target the appropriate breeding strategies and act as a guide to optimize and modernize the rice breeding program for accelerated genetic gains. The rate of genetic gain in the IRRI’s rice drought breeding program has never been estimated. On the other note, the historical or current elite breeding lines are an important genetic resource that can be directly used in the population improvement-based breeding programs to improve the genetic gains. Further, integrating the modern genomic tools and technologies with the population improvement-based breeding programs using elite lines as a genetic resource will boost the genetic gains (Xu et al. 2017). However, it is important to select the appropriate lines from the historical breeding pool representing the overall genetic diversity in the breeding pool and should have high mean performance for grain yield and possess the major genes or haplotypes for mendelian traits.

Thus, to assess genetic gains in the IRRI’s rice drought breeding program and select the valuable elite lines as a future genetic resource, this study was conducted to (1) estimate the genetic trends for grain yield in IRRI’s rice drought breeding program by leveraging 17 years of historical data from the advanced yield trials (AYT) managed under drought and non-stress conditions and, (2) identify high yielding drought-tolerant lines based on the grain yield breeding values as a future genetic resource for recurrent selection program. The AYT trials included premium released varieties from IRRI globally, therefore, are the potential reservoirs to select some of the high-yielding drought-tolerant lines as a future genetic resource.

## Materials and Methods

### Description and Pre-processing of Historical Data

For this study, historical data from yield trials conducted under non-stress and drought conditions (at the reproductive stage) at IRRI, Philippines from 2003 to 2019 (17 years) were used. The trials in each year were conducted in two seasons-dry (from January to April) and wet (from late June to September) season. For all the trials, drought stress was implemented at the reproductive stage (panicle initiation stage) by draining the field completely. The PVC pipes were used and installed a week before stress imposition to help keep a track on the water table depths in the fields. The complete management of the drought stress and non-stress breeding trials at IRRI is detailed by Venuprasad et al. (2009).

The combination of year, season, and treatment were treated as a trial or environment. In total, the historical data harbored 19,916 data points with 2497 unique lines. The data was pre-processed, and the quality of phenotype records was checked initially to ensure high-quality trials and phenotypes are retained for downstream analysis. The data was checked for extreme or unexpected values, missing percentages, and valid experimental design. The trials having more than 20% of missing data for grain yield, lack of replications or proper experimental design were dropped initially. Further, the extreme observations were checked in the data before outlier detection as they may increase the error variance, which may affect the performance of the outlier detection (Gonzalez et al. 2018). After preprocessing, data were checked for outlier detection using the Bonferroni-Holm test for studentized residuals test (Bernal-Vasquez et al. 2016; Philipp et al. 2019). The 88 data points detected as outliers were removed from the data to make sure only high-quality data points are retained for reliable estimates. In total, 53 trials harboring 19,828 data points with 2490 unique lines were retained for downstream analysis. The complete information on the trials, including the year, season, treatment, experimental design, number of plots, replications, and blocks, are provided in the Additional file 1: Table S1. Trials were performed in varied experimental designs including alpha lattice, augmented randomized complete block, and randomized complete block designs (RCBD). Three major agronomic traits days to 50% flowering (DTF), plant height (PH), and grain yield (kg/ha) were retrieved and used for downstream analysis.

### Extraction of Pedigree

The pedigree data of 2490 unique lines were extracted from the breeding data management system (Breeding 4 Results (B4R), 2021, https://b4r.irri.org) which has the passport to comprehensive information of the genotype, phenotype, and pedigree data of breeding lines. Additional information on the parents and grandparents up to seven generations, cross-type for each line, and breeding strategy were extracted using the IRRI genealogy management system (McLaren et al. 2005; Collard et al. 2019) using customized R scripts. These advanced breeding lines were bred utilizing various crossing strategies including single, double, three-way, complex crosses and backcrosses.

### Statistical Modelling of Phenotypic Data

A two-stage approach of mixed model analysis was used to analyze the data and extract the breeding values for grain yield, DTF, and PH (Piepho et al. 2008, 2012; Smith and Cullis 2018) under non-stress, drought, and by analyzing drought and non-stress together. The two-stage approach was adopted to account for different experimental designs across the environments (Damesa et al. 2017). In the first stage, per year adjusted means as BLUEs for each genotype were estimated in each environment. The mixed model used consisted of genotypes as fixed effects, and season replications and or blocks were used as random effects. BLUEs for each genotype per year was obtained using the following linear mixed model:

1 $$y_{ijkl} = \mu + g_{i} + r_{j} + b_{k} + s_{l} + \varepsilon_{ijkl }$$

where, ${y}_{ijkl}$ represents adjusted means for *i*th observation in *j*th replication, *k*th block and *l*th season, μ is the overall mean, g_i_ is the fixed effect of *i*th genotype, r_j_ is the random effect of replications in each trial, b_k_ is the random block effect, s_l_ is the random effect for season and ${\varepsilon }_{ijkl}$ is the residual error. The random effects were distributed independently and identically. In this model, DTF was used as a covariate for reducing the error on yield caused due to the presence of different maturity genotypes. Further, the present study focused on the effect of drought on the grain yield during the reproductive stage, thus excluding the impact of drought on the vegetative growth stage.

The above model was used for the trials which were performed using an alpha-lattice breeding design. For environments with augmented RCBD experimental design, replications were considered equal to blocks, and hence block effect was removed. Likewise, BLUEs and standard error values were calculated respectively for each genotype per year for the DTF and PH traits using Eq. 1.

The combined analysis using a linear mixed model was used to extract the single value BLUEs adjusted across the non-stress and drought treatments. The model used follows as:

2 $$y_{ijklm} = g_{i} + r_{j} + b_{k} + s_{l} + t_{m} + \varepsilon_{ijklm}$$

all the terms are described in Eq. 1 except the ${t}_{m}$ which is the fixed effect of *m*th treatment (non-stress and drought). We assume different variances across non-stress and drought treatments in the model to get the adjusted means.

Heritabilities for grain yield in each environment across non-stress and drought conditions were calculated for each environment (Cullis et al. 2006; Piepho and Möhring 2007). The same model described above was used to calculate the heritability with genotypes as a random effect. The equation to calculate heritability follows as:

3 $$H^{2} = 1 - \frac{{\overline{V}_{BLUP} }}{{2\sigma_{g}^{2} }}$$

where ${\overline{V} }_{BLUP}$ is the mean–variance difference of two BLUPs and σ^2^g is the variance of genotypes. It is important to note that the above equation used to calculate heritability may not be appropriate if the data is highly unbalanced. However, in our case, the unbalancedness in data was not so high as we estimated the heritability per trial, i.e., each growing season in each year.

In the second stage analysis, a pedigree-based mixed model approach was used to extract the breeding values each in non-stress, drought, and combined data. Additionally, the BLUE’s estimated from the first stage analysis were weighted by the inverse of their squared standard errors and used as response variable in the second stage analysis (Möhring and Piepho 2009). The BLUEs subjected with weights were used to take care of the heterogeneous error variances. The model used was as follows:

4 $$y_{ij} = \mu + g_{i} + ye_{j} + \varepsilon_{ij}$$

where ${y}_{ij}$ is the BLUE values implied with weights for *i*th observation in *j*th year, μ is the overall mean, ${g}_{i}$ is the breeding value of *i*th genotype with g_i_ ∼ N (0, Aσ^2^_g_) where σ^2^_g_ is the genetic variance and A is the additive genetic relationship matrix based on pedigrees, ${ye}_{j}$ is the random effect of year, and ${\varepsilon }_{ij}$ is the residual error, with ${\varepsilon }_{ij}$ ∼ N(0, Rσ^2^_ε_), where R is the identity error covariance matrix and σ^2^_ε_ is the error variance. The reliability of the breeding values (Isik et al. 2017) of each genotype was estimated using the following equation:

5 $$r = 1 - \frac{PEV}{{\sigma_{g}^{2} }}$$

Two-stage mixed model data analysis was performed in the R software (R Core Team 2020) using the ASReml-R 4 package (Butler et al. 2017). The R package AGHMatrix was used for constructing the pedigree A-matrix (Amadeu et al. 2016). The analytical pipeline and codes are available on the GitHub (https://github.com/whussain2/Genetic_Trend_Rice_Drought).

### Estimation of the Genetic Trends

The genetic gain was estimated separately for three conditions: (a) non-stress trials, (b) drought trials, and (c) combined data (adjusted means across non-stress and drought trials). For the genetic gain trend, breeding values were regressed on the year of origin of the line. The genetic trend was also estimated for released varieties and checks by regressing the breeding value of checks on the year of origin in non-stress trials, drought trials, and combined data.

### Identification of Breeding Panel

Breeding values obtained by combined analysis of non-stress and drought data were used for the identification of elite genotypes as a future breeding resource. A total of 200 lines were selected from the 2490 unique historical lines based on the higher breeding values and prediction accuracy of > 0.4. In addition to the lines with higher breeding values and reliabilities for grain yield, lines harboring the key QTLs responsive for various biotic and abiotic stresses were also selected for formulating the breeding panel. To make sure genotypes selected are diverse and represent the whole collection of lines in historical lines, the pedigree matrix was used in the analysis to account for similarity among the lines. The similarity and diversity among the selected lines in comparison to the whole collection were visualized through bi-plot. For bi-plot, principal component analysis (PCA) was performed on the pedigree-based relationship matrix using the *princomp()* function in R software. Bi-plot was visualized using the *factoextra* R package (Kassambara and Mundt 2017).

## Results

### Descriptive Features of Historical Drought Data

**Fig. 1:**
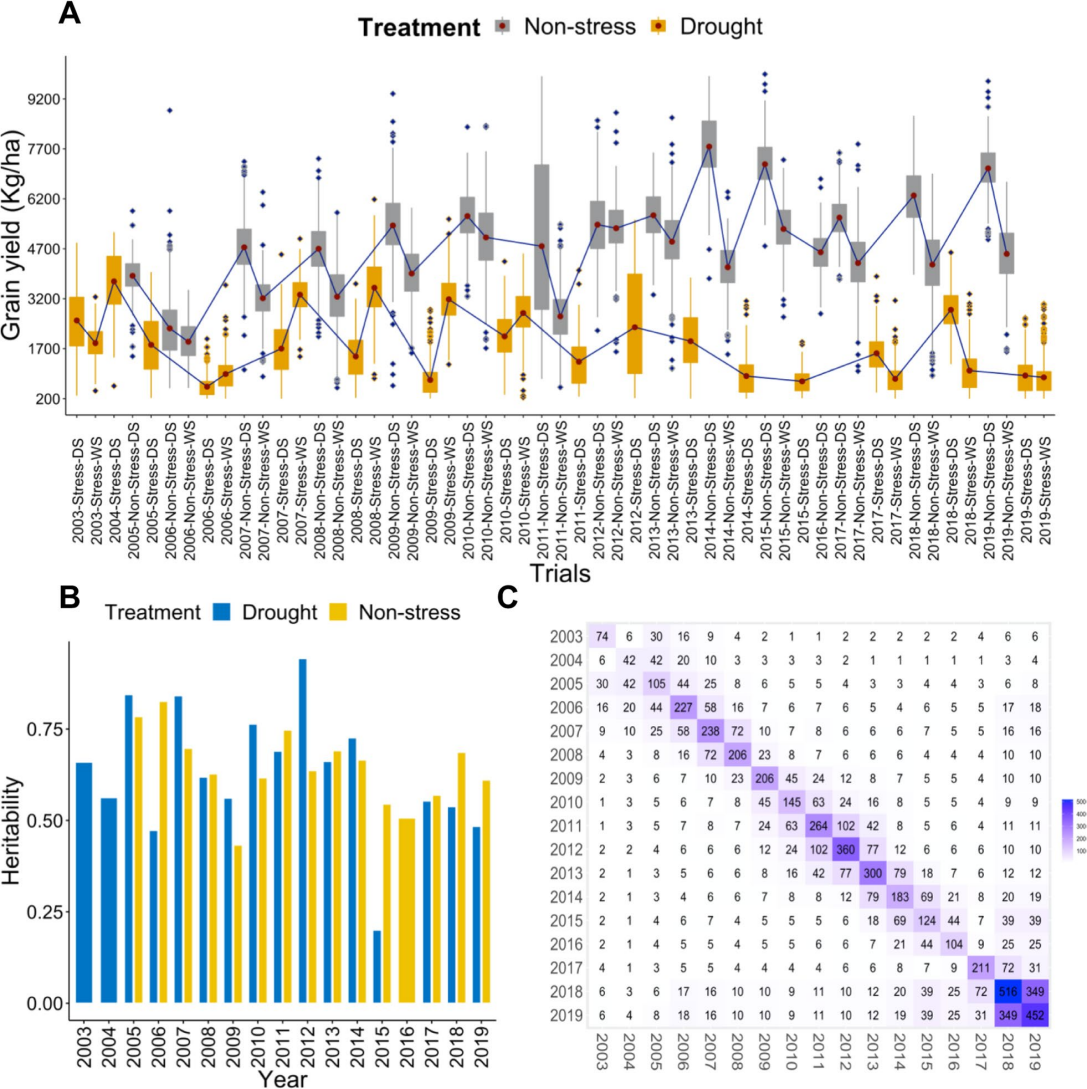
**a** Boxplot showing the raw mean grain yield (kg/ha) under non-stress and drought conditions from the year 2003–2019. The x-axis shows the trial names, which are combinations of year, season, and growing condition. In the boxplots, it is clear the grain yield is higher under non-stress conditions as compared to drought conditions indicating the impact of drought on the yield trials. **b** Heritabilities of the trials in each year from 2003 to 2019. The blue bars represent drought and yellow non-stress trials. **c** Connectivity of all the genotypes across years from 2003 to 2019. The genotypes including common checks and promising varieties were repeatedly tested for their performance in the successive years, thereby making the dataset well connected across successive years. The numbers in the boxes show the genotypes that were common between years

The three main traits grain yield, PH, and DTF grown under non-stress and drought conditions were used for analysis. The difference in phenotypic trait values for all three traits was observed in non-stress and drought conditions. The difference in trait value was also evident across the years. The raw mean grain yield under the non-stress conditions ranged from 514.40 to 9943.80 (kg/ha) and under the drought conditions 201.10–6181.21 (kg/ha) (Fig. 1a). In each trial, lower yield values were observed under drought conditions as compared to trials under non-stress conditions indicating the impact of drought on the phenotypes. The DTF values under the non-stress conditions ranged from 60 to 119 days and in the drought conditions it ranged from 63 to 129 days (Additional file 2: Figure S1). Further, we observed a wide distribution in DTF for the genotypes, and genotypes based on breeding values were classified into three maturity groups based on the breeding values i.e., early (85–109 days), medium (110–124 days), and late (DTF ≥ 125 days). The DTF of 68% of the lines from the complete unique set of lines under drought implication falls into the medium duration maturity category. The remaining genotypes make up 15% and 16% for early and late duration groups, respectively. Similarly, under the non-stress conditions, the percentage of lines falling into three maturity categories were, early (15%), medium (76%), and late (9%) respectively. Because of the wide distribution in DTF, we used DTF as covariate in the phenotypic data modeling to adjust for the grain yield. Similarly, for PH we observed a wide distribution in phenotypic values and PH in the data set ranged from 40 to 195 (cm) under non-stress conditions and 40–90 (cm) under drought conditions (Additional file 2: Figure S2). The dataset has ample diversity among the tested genotypes having a diverse range for PH between 40 and 195 cm. Low plant height was observed under drought conditions, consistent with the previous reports (Ahmadikhah and Marufinia 2016; Mishra and Panda 2017; Hussain et al. 2018; Panda et al. 2021). Heritability for grain yield estimates ranged between 0.20 and 0.94 under the drought trials and 0.43–0.83 under non-stress growing trials for the non-stress trials (Fig. 1b). Out of 17 trials, 8 trials showed lower heritability values under drought conditions as compared to non-stress trials. Reduction in heritability under drought conditions is a common phenomenon (Henry et al. 1997; Kumar et al. 2007) which indicates that genotypes are not able to express the higher genetic potential for grain yield.

### Historical Data Connectivity

Historical data sets usually have very low connectivity as new lines are being tested every year, and only a limited number of times the new lines are being tested. In the current data set, we observe appropriate connectivity of the different genotypes across the years (Fig. 1c), and this connectivity was mainly created by long-term checks (IR64, Swarna, Sahbhagi Dhan, IRRI 154) across the years. Further, over the breeding cycles and years, the superior genotypes were forwarded and re-tested in the succeeding years which made the dataset well connected to previous years (Additional file 2: Figure S3). Further to ensure good connectivity and get reliable estimates of the breeding values, relationship matrix (Additional file 2: Figure S4) based on pedigrees of 2490 unique lines was incorporated in the second stage of mixed model analysis. Additionally, during the analysis, the trials were separated into two growing conditions (non-stress and drought), and seasons (wet and dry), good connectivity across seasons and growing conditions was observed among the lines (Additional file 2: Figure S5).

### Estimation of Breeding Values

Breeding values obtained from second-stage analysis by fitting a pedigree matrix were used to estimate the genetic trends and used to identify the best lines based on higher breeding values for the formulation of the core panel. The range of the breeding values for the genotypes under drought stress was between 642.79 and 3267.60 (kg/ha). Under the non-stress growing conditions, the breeding values of the genotypes ranged between 3447.93 and 6933.32 (kg/ha). The breeding values adjusted across drought and non-stress growing conditions ranged between 1026.93 and 4622.59 (kg/ha). The histogram of the breeding values along with the mean and standard deviations is given in the (Additional file 2: Figure S6).

### Estimation of Genetic Trends

**Fig. 2:**
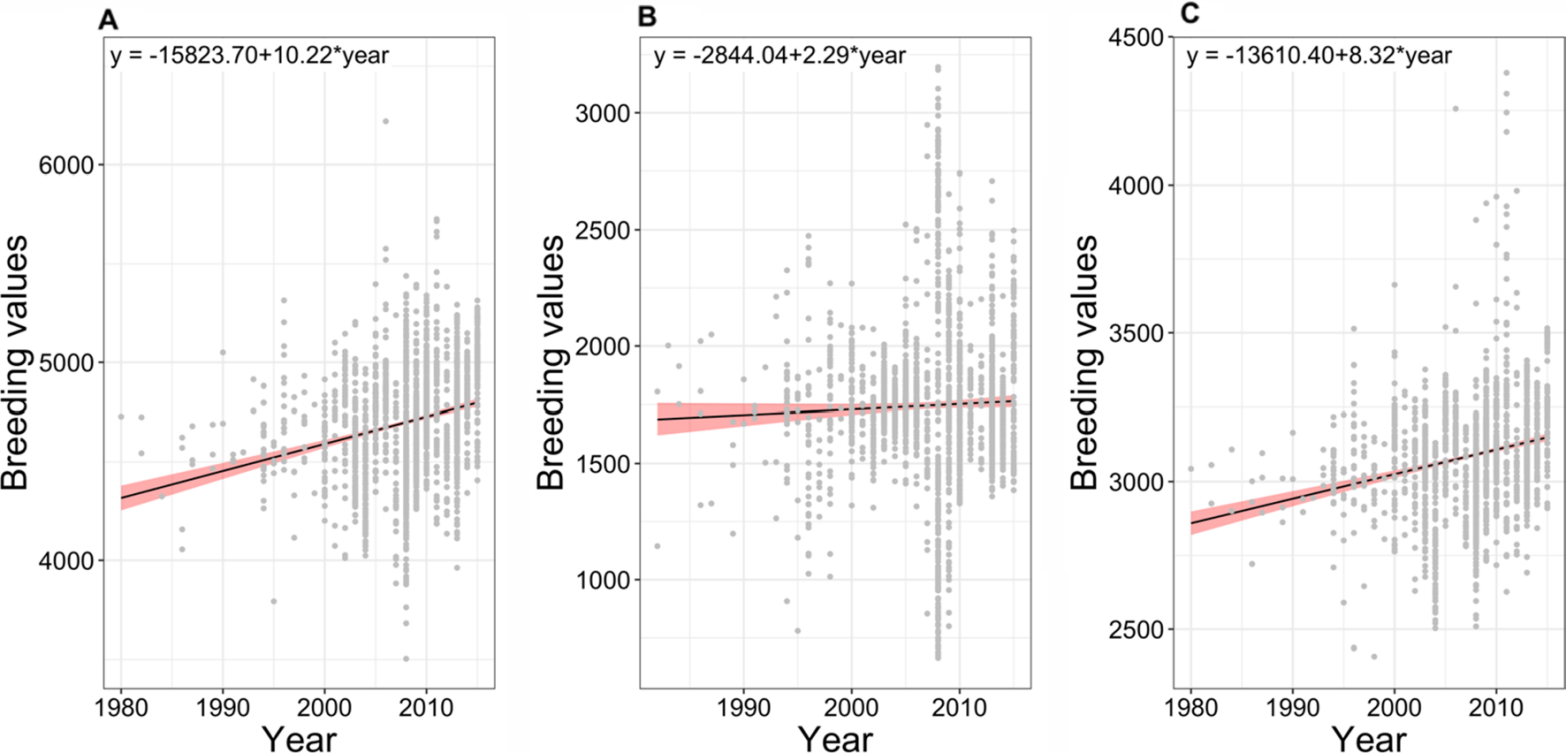
Trends in genetic gain from IRRI’s 17 years of drought breeding program under. **a** Non-Stress conditions, **b** drought conditions, and **c** combined conditions (adjusted breeding values under drought and non-stress conditions). The x-axis shows the year of origin of the genotype ranging from 1980 to 2015 and the y-axis shows the breeding value of the genotype. The genetic gain was estimated by regressing the breeding values of grain yield on the year of origin of genotype and is given by the slope of the line

**Fig. 3:**
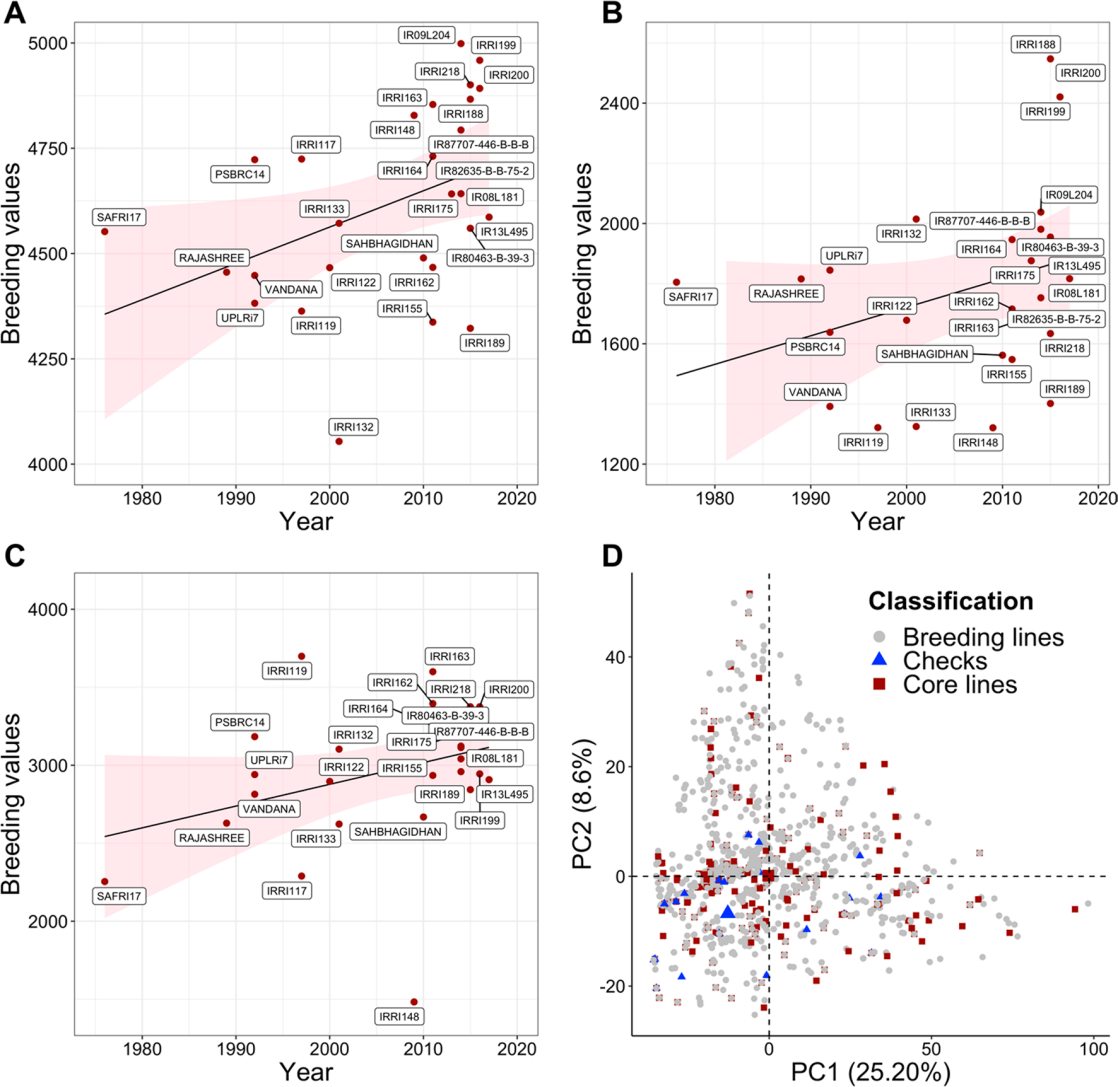
Trends in genetic gain for IRRI drought released lines and popular checks under three conditions: **a** non-stress, **b** drought and **c** combined conditions. The genetic gain was estimated by regressing the breeding values on the year of origin of genotype, **d** shows the biplot of the lines selected based on the breeding value for grain yield as an elite core panel. Core panel lines are highlighted in dark red color. The checks and released lines are shown in blue color, and the whole historical collection lines are represented in gray color. The biplot was constructed using the first two principal components obtained from the pedigree-based relationship matrix. The selected lines represent and capture the variability of the whole collection of genotypes and are ideal to form the core panel as a future breeding resource

The genetic trend was estimated for the genotypes and, also for checks and released varieties in non-stress growing conditions, drought conditions, and in combined data. Under non-stress growing conditions, the genetic gain of 0.21% with a yield advantage of 10.22 kg/ha per year was observed for genotypes (Fig. 2a), and genetic gain of 0.17% was observed for checks and released lines representing an increase of 7.90 kg/ha per year (Fig. 3a). The genetic trend under the drought conditions exhibited a positive trend with a genetic gain of 0.13% for genotypes (Fig. 2b) and 0.55% for released lines (Fig. 3b). Yield advantages of 2.29 kg/ha for genotypes and 9.52 kg/ha for checks was observed. The regression estimates for combined analysis (adjusted breeding values across non-stress and drought growing conditions) showed a genetic gain of 0.27% for genotypes (Fig. 2c) and 0.60% (Fig. 3c) for checks with a yield advantage of 8.32 kg/ha for genotypes and 13.69 kg/ha for checks. Further genetic gain trends were plotted using non-parametric approach with the method Local weighted regression or *loess* to get information on the short-term and long-term genetic trends in the breeding program (Additional file 2: Figure S7). We correlate the genetic trends with the breeding strategies adopted by the drought breeding program at IRRI. The breeding strategies used by rice breeders to breed for drought tolerance ranged from single seed descent breeding based on single or complex crosses to backcross marker assisted breeding for the introgression of various trait related QTLs.

### Comparison of Breeding Values

We compared the breeding values of the popular checks and the released varieties from IRRI, to assess their performance under three conditions viz*.,* stress, non-stress, and combined drought and non-stress. This assessment was undertaken with the overview to deduce the best performers among the released IRRI lines. The performance of the released varieties was superior to the popular checks under all three conditions (Fig. 3a–c). The varieties IRRI 188, IRRI 199, and IRRI 200 had superior breeding values of 2547.19 (kg/ha), 2420.70 (kg/ha), and 2490.97 (kg/ha), respectively, compared to popular checks and other released varieties when estimated under the drought stress conditions (Fig. 3a). These varieties were released in the years 2015 and 2016, depicting growth of the breeding program over the preceding released varieties developed in the previous years. The latter also showed the higher performance to the most popular drought-tolerant released variety Sahbhagi Dhan with the predicted value of 1562.03 (kg/ha) under stress conditions. The breeding values for popular drought-tolerant checks Vandana and Rajashree were 1391.68 (kg/ha) and 1815.65 (kg/ha) under drought stress conditions respectively. This depicts the superiority of the recently released lines over popular checks and varieties. The genotypes were also assessed for the trials evaluated under non-stress conditions. The breeding lines, IR09L204 depicted the highest breeding value of 4998.44 (kg/ha) followed by IRRI 199 (4958.74), IRRI 218 (4900.64), IRRI 200 (4892.13), and IRRI 188 (4866.45) in the descending fashion of the superior performing genotypes under the non-stress conditions (Fig. 3b). In the combined analysis with breeding lines performing superior under both drought stress and non-stress conditions were as follows IRRI 119 (3698.75), IRRI 163 (3599.56), IRRI 162 (3395.07), IRRI 218 (3374.91), and IRRI 200 (3374.93) (Fig. 3c). Similar to drought conditions, these lines surpassed popular checks Vandana (2814.09) and Rajashree (2628.67); variety Sahbhagi Dhan (2668.12). The breeding values of the five top-performing lines have been detailed in Fig. 3a–c for non-stress, drought, and combined conditions, respectively.

### Identification and Development of Elite Breeding Panel

The genotypes with higher breeding values (obtained from the combined analysis of non-stress and drought data) based on the grain yield and with prediction accuracy of > 0.40 were selected for the development of the breeding core panel. In total 200 promising lines were identified and used for the development of the elite panel. To make sure 200 selected lines are genetically diverse and representative of decades of IRRI’s drought breeding and varietal development, a pedigree matrix was used to account for genetic similarity among the genotypes. The relationship matrix constructed using pedigree data was visualized using the biplot (Fig. 3d). From the biplot, it is very clear how diverse the selected lines are, and how they represent and capture the diversity of the whole historical collection of 2490 unique genotypes. The breeding values of the 200 genotypes formulating the core panel ranged between 3200 and 4622.59 kg/ha. The mean breeding value of the panel is 3395.10 with a standard deviation of 373.23 (Additional file 2: Figure S8).

## Discussion

Here we provide an overview of how 17 years of historical IRRI’s rice drought data was leveraged to estimate the breeding values for grain yield and estimate the genetic trends in the IRRI’s rice drought breeding program. We also demonstrated how the top-performing lines based on the grain yield breeding values were selected as the future breeding elite resource for recurrent selection. Recurrent selection type breeding strategy could be combined with the genomic prediction to shorten the breeding cycle, increase selection accuracy and intensity, thus increasing the rate of genetic gain (Zhang et al. 2021).

For the selection of genotypes as a part of the core panel, pedigree information availability was pivotal in fitting the additive matrix in the second stage of the mixed model analysis for reliable estimation of the breeding values and help in the selection of accurate genotypes for the formulation of the core panel. The essence of using the relationship matrix is that it contains information about the flow of genes and explicitly allows the dissection of genetic variation by accounting for the additive genetic covariances between random effects/genotypes for reliable estimation of the breeding values (Piepho et al. 2008). Further, the relationship matrix ties up the data across years by borrowing information from parents and grandparents and creating the connectivity in the highly unbalanced data set for the reliable estimation of breeding values.

### Genetic Trend and Breeding Value Estimations

Improving the crop yield or genetic gain is crucial and can be attained by the breeders via implementing data driven breeding (Xu et al. 2017). The assessment of genetic gains to estimate crop yield growth has had a limited focus in the past. However, genetic gain estimation with an outlook to reinforce the future breeding programs to increase genetic gain for yield has become a major focus. In this study, the regression of breeding values over the year of release/testing indicated a positive genetic gain under all three conditions, and the overall success of the drought breeding program at IRRI. However, the genetic gain of 0.13% observed under drought conditions is not sufficient to meet the current and future rice food demands. A much higher genetic gain of 1.9% was reported under severe drought conditions at the reproductive stage in rice evaluated in IRRI India (Kumar et al. 2021). Therefore, it is essential to optimize and modernize the drought rice breeding program at IRRI for enhanced genetic gains.

Further we observed a minimal increase in genetic gain till the 2005 as the year of origin of line, post which there was higher and constant increase in genetic gain in all the three conditions (Additional file 2: Figure S7). The increase in genetic gain in the latter stages could be accredited to differential breeding strategies followed in the drought breeding program across the years. The drought breeding program at IRRI until 2008 was led specifically for targeting the introgression of major abiotic stress-tolerant QTLs/genes for the development of NILs possessing elite genetic backgrounds with minimal focus on recurrent selection breeding strategy. Thereafter, in the preceding years, the focus drifted towards pyramiding these genes/QTLs for rendering multiple stress-tolerant cultivars. These genotypes were not precisely targeted for genetic gain enhancement; however, their genetic merit is highly valuable as these sustainable varieties were disseminated for commercial cultivation across countries and can withstand additional biotic and abiotic stresses along with drought. Further in the last few years more emphasize was given to recurrent selection-based breeding strategies in the drought breeding program to improve the yield and increase genetic gain. Rather than the QTL-based introgression or breeding approach as used previously, rice breeders need to focus on population improvement breeding approaches using elite lines as parents, wherein parents of each breeding cycle are selected based on high additive breeding values for the grain yield. Recurrent selection schemes focused on quick recycling of the best and high breeding value lines may deliver higher rates of genetic gain (Cobb et al. 2019). Further to ensure the constant genetic gains, rice breeders need to select the parents for new breeding cycles that have higher additive breeding value than the previous breeding cycle. However, for long-term genetic gains, utmost care must be taken by the breeders to diversify the elite gene pool by bringing or directly crossing the exotic or diverse materials with elite pool lines. Exotic or diverse materials may broaden the elite gene pool, but they are highly unadopted and unimproved lines with lower breeding values. Thus, crossing a directly diverse line with an elite line may bring novel favorable alleles and increase the genetic variance of progeny, however, they barely counterbalance the mean performance of progeny due to the low breeding value of diverse or exotic lines (Longin and Reif 2014; Allier et al. 2020). Thus, a focused recurrent selection breeding approach with a systematic pre-breeding approach is required to deliver higher and constant genetic gains in IRRI’s drought rice breeding program.

The drought breeding program at IRRI has successfully released many drought-tolerant varieties across Asia and Africa (https://strasa.irri.org/), and most of them were part of this study. We separately assessed the genetic gain estimation for released varieties, and higher genetic gain was observed for varieties/checks released for use under drought conditions. The positive and higher genetic gain (0.55%) for checks under drought indicates that a strong impact has been made by IRRI’s drought breeding program to increase rice productivity under challenging and extreme environments. Among these varieties- IRRI 188, IRRI 199, and IRRI 200 released during 2015 and 2016 had higher breeding values as compared to the popular checks Rajashree, Vandana, and UPLRi7, and previously released varieties. The superiority of these lines over the formerly released varieties indicates growth in grain yield to a larger extent. Also, positive genetic trend and higher breeding values of the recently released varieties demarcates the positive growth of the breeding program across years as the performance improved from the preceding released varieties developed in the previous years. Further, among these three top-performing released lines, IRRI 199 originates from a backcross breeding program utilizing a tropical japonica drought and blast tolerant genotype, Moroberekan, as a donor parent and a high-yielding, semi-dwarf Indica rice variety, Swarna. The population harbors a major, severe drought-tolerant QTL, *qDTY3.2* contributing to various drought-tolerant traits viz*.,* canopy cover, canopy temperature (CT), root system architecture (RSA) attributes (Wasaya et al. 2018; Sofi et al. 2019). The genetic region co-localizes with early flowering time QTL *HD9* and lodging resistance features, making the genotype suitable for various ecosystems and environments (Dixit et al. 2014, 2015). Concomitantly, based on the combined analysis IRRI 163, IRRI 162, IRRI 218 and IRRI 200 released in the years 2011, 2015, and 2016 exhibited high breeding values under both drought and non-stress conditions.

### Development of Breeding Panel as the Future Breeding Resource

In the last 1-decade rice breeders at IRRI have mainly focused on introgression and pyramiding of major abiotic stress-tolerant QTLs/genes in elite backgrounds (Venuprasad et al. 2009; Mishra et al. 2013; Yadaw et al. 2013). Population improvement based on recurrent selection and early re-cycling of advanced lines has not been a major focus of the drought rice breeding program at IRRI. Different crossing strategies (Additional file 2: Figure S9) single, complex, double, and backcrosses have been used by the rice breeders to integrate these QTLs into the elite genetic backgrounds and develop the new breeding lines. Diverse materials, including landraces, and donors have been extensively used to diversify the gene pool and develop climate-resilient varieties (Sandhu et al. 2021; Yadav et al. 2021). However, this strategy of diversifying the elite gene pool with limited focus on recurrent selection and early re-cycling of high-value breeding lines may have limited genetic gain to a large extent and has not been sufficient to maintain the higher genetic gains over time.

Recurrent selection with early re-cycling of lines is the key to increase the frequency of desirable additive haplotypes of grain yield in each cycle, and ultimately boosting the genetic gains. To strictly focus on recurrent selection breeding schemes, the presence of highly characterized elite lines with higher breeding values for grain yield and possessing the key haplotypes for mendelian traits is required as the base population. The historical data set used in this study which contains 2497 unique genotypes has been used by rice breeders at IRRI for decades and in the past 60 years, many promising drought lines have been extracted from this breeding pool. This breeding pool exhibits ample genetic diversity and possesses the key lines that may be used as a future breeding resource to sustain higher grain yield under challenging environments. Further, this breeding collection has not only been improved for grain yield but also turbocharged with diverse alleles for important traits of biotic and abiotic stresses and represents the overall diversity and breadth of IRRI’s rice drought breeding program. Besides the gene bank resources, this breeding pool represents the important source of genetic variation that is highly dynamic created through recombination and reshuffling of alleles. Thus, identifying the high-power performing lines based on the breeding values for grain yield that represents the overall diversity of the whole breeding collections is the key to success in future recurrent selection breeding strategies. To this end, we took this initiative to extract the top breeding lines from the whole historical breeding collection and form the elite breeding panel as future breeding resources. These selected lines besides possessing high breeding values are also indicative of higher recovery capabilities under drought stress. We believe the lines selected are the best genetic variation to recombine and reshuffle in recurrent selection to increase the frequency of additive haplotypes of grain yield in each cycle of breeding. Further, the recurrent selection type breeding strategy could be combined with the genomic prediction tool to select the lines based on genomic estimated breeding values. Genomic prediction implemented in earlier generations will also help to decrease the cycle length. Further, selection on more candidates in earlier or advanced generation can be carried out using genomic predictions, thus increasing the rate of genetic gain (Zhang et al. 2021).

Additionally, based on the comprehensive review of the literature, we assessed the additional characteristic features of these selected lines and found these selected lines harbor favorable QTLs for resistance to biotic stresses (bacterial blight, blast, brown planthoppers, stemborer, whiteheads, green leafhoppers, and rice tungro virus), drought tolerance, and quality traits (Additional file 1: Table S2). It is evident from the table that these lines are turbocharged with key QTLs and is a readily available elite genetic resource for future recurrent selection breeding schemes for targeting preferred environments/countries based on the desirable market profiles. However, we emphasize the detailed genotypic characterization of these lines in the future to extract more information for their efficient use in drought breeding program.

Additionally, the elite panel also harbored two best performing genotypes; IR15F1706 and IR 54447-3B-10-2 from the 2020 drought stress trials (unpublished) showing high chlorophyll fluorescence (CF) and low CT values. These genotypes when assessed had high breeding values, confirming further the accuracy of the analysis undertaken. Alongside, it also demarcates that deprivation in CT has a strong influence on plant’s yield under drought-prone conditions. It has also been reported that CT has a high correlation with the RSA traits symbolizing enhanced genetic capacity of the plant to retain soil moisture and hence improved survival and yield under drought stress conditions (Blair et al. 2010; Lopes and Reynolds 2010). Similarly, enhanced CF demarcates a plant's capabilities to withstand drought stress effectively. Furthermore, two multiparent conventional bred lines namely, IR 115844-B-B-281-1-2 and IR 115844-B-342-1-1-1 present in the selected panel have been reported to yield more than 7000 kg/ha under dry direct-seeded conditions, with higher yields under non-stress and reproductive stage drought stress conditions (Sandhu et al. 2021). In our study, these lines showed the breeding values between 3200 and 3368.14, which form a valuable resource for the breeding programs to be utilized for drought-prone areas with major cultivation under dry seeded conditions having limited water and labor availability. Few of the top 100 selected lines were also reported by other research studies to show higher yield performance under multi-environment trials (Vergara et al. 2016). Furthermore, we believe that the increase in the breeding values of the lines under drought conditions is mainly due to the introgression of the major drought QTLs.

In summary, the core breeding panel selected based on the breeding values and prediction accuracy is an important genetic resource possessing multiple stress tolerance, varied range of quality traits with genotypes suited for cultivation under challenging environments. Furthermore, they form an easily available and highly enriched genetic resource for future recurrent selection programs and enhance genetic gains. However, we emphasize systematic genotypic and phenotypic characterization of these lines in achieving more knowledge on the value proposition of these lines, new allele enrichment, and help to create a framework for better understanding and managing the genetic diversity in the elite pool. However, the question may arise whether continued use of the elite pool lines and reshuffling of alleles in closed recurrent selection strategies is enough to maintain long-term genetic gains? Most will agree with the enrichment of the elite breeding pool with diverse materials as was done previously in the IRRI’s drought breeding program. However, we emphasize here a systematic effort to diversify the elite gene pool without contaminating it with diverse materials and limiting the genetic gains (Allier et al. 2020).

## Conclusions

The drought breeding program at IRRI has been successful in maintaining a positive genetic rate in the breeding program, however, the increase in genetic gain has not been so high to fulfill the rice food demands. To achieve the required genetic gains of 1.5% or above, a recurrent selection breeding strategy of the elite population with the integration of modern tools and technologies is needed. Genotypic and phenotypic characterization of the selected elite panel is required to effectively manage, incorporate, and track the genetic diversity for short-term and long-term genetic gains. Further, efficient pre-breeding strategies are needed to turbocharge the elite gene pool with major haplotypes of traits showing discrete Mendelian segregation without compromising the performance of elite lines and boost the genetic gains.

## Supplementary Information

**Additional file 1**. **Table S1.** List of trials used in this study for genetic trend estimations and formulation of elite core panel. **Table S2.** Delineation of the traits and characteristics of top-performing genotypes formulating the breeding panel.

**Additional file 2**. **Figure S1.** Boxplots showing distribution of raw data values for days to 50% flowering (DTF) data under non-stress and drought conditions. **Figure S2.** Boxplots showing the distribution of raw data values for plant height (cm) data under non-stress and drought conditions. **Figure S3.** Depiction of the number of same genotypes tested across the years in the breeding program. **Figure S4.** Pedigree-based heat map and clustering of the genotypes bred over the years. **Figure S5.** Heatmaps showing connectivity of lines across different growing conditions and seasons. **Figure S6.** Distribution of the breeding values for grain yield. **Figure S7.** Shows genetic trends using a non-parametric approach based on method loess under a) under non-stress conditions, b) drought, and c) combined conditions. **Figure S8.** Distribution of 200 selected genotypes for grain yield breeding values. **Figure S9.** Breeding schemes implemented each year in the drought breeding program from 2003–2019.

## Data Availability

The datasets included in this work are given as additional files. The additional data and scripts used to run the analysis can be found on the GitHub page at the following link: https://github.com/whussain2/Genetic_Trend_Rice_Drought.

## Abbreviations

BLUP: Best linear unbiased predictions
BLUE: Best linear unbiased estimates
REML: Residual maximum likelihood
GEBV: Genomic estimated breeding values
CV: Coefficient of variation
AYT: Advanced yield trials
PH: Plant height
DTF: Days to 50% flowering
PCA: Principal component analysis
QTL: Quantitative trait loci
MABB: Marker-assisted backcross breeding
RSA: Root system architecture
