# Supplementary material for: Genetic Trends Estimation in IRRIs Rice Drought Breeding Program and Identification of High Yielding Drought-Tolerant Lines
Source: Rice (N Y). 2022 Mar 5;15:14. doi: 10.1186/s12284-022-00559-3 (PMC8898209; doi:10.1186/s12284-022-00559-3)
Supplement: Supplementary file 2 — Additional file 2. Figure S1. Boxplots showing distribution of raw data values for days to 50% flowering (DTF) data under non-stress and drought conditions. Figure S2. Boxplots showing the distribution of raw data values for plant height (cm) data under non-stress and drought conditions. Figure S3. Depiction of the number of same genotypes tested across the years in the breeding program. Figure S4. Pedigree-based heat map and clustering of the genotypes bred over the years. Figure S5. Heatmaps showing connectivity of lines across different growing conditions and seasons. Figure S6. Distribution of the breeding values for grain yield. Figure S7. Shows genetic trends using a non-parametric approach based on method loess under a) under non-stress conditions, b) drought, and c) combined conditions. Figure S8. Distribution of 200 selected genotypes for grain yield breeding values. Figure S9. Breeding schemes implemented each year in the drought breeding program from 2003–2019. [file 12284_2022_559_MOESM2_ESM.docx]

Supplementary Information

**Genetic Trends Estimation in IRRIs Rice Drought Breeding Program and Identification of High Yielding Drought-tolerant Lines**

Apurva Khanna^1^, Mahender Anumalla^1^, Margaret Catolos^1^, Jerome Bartholome^2^, Roberto Fritsche-Neto^1^, John Damien Platten^1^ Daniel Joseph Pisano^1^, Alaine Gulles^1^, Ma Teresa Sta. Cruz^1^, Joie Ramos^1^, Gem Faustino^1^, Sankalp Bhosale^1^, Waseem Hussain^1^*

^1^Rice Breeding Innovation Platform, International Rice Research Institute (IRRI), Los Banos, Laguna, Philippines

^2^CIRAD, UMR AGAP, Montpellier, France, AGAP, Univ Montpellier, CIRAD, INRA, Montpellier SupAgro, Montpellier, France

*** Corresponding author:**

**Waseem Hussain**

International Rice Research Institute (IRRI), Los Baños, Laguna 4031, Philippines,

email: [waseem.hussain@irri.org](mailto:waseem.hussain@irri.org)

**Additional file 1: Table S1**: List of trials used in this study for genetic trend estimations and formulation of elite core panel.

**Additional file 1: Table S2**: Delineation of the traits and characteristics of top-performing

genotypes formulating the breeding panel.

**Additional file 2: Figure S1**: Boxplots showing distribution of raw data values for days to 50% flowering (DTF) data under non-stress and drought conditions.

**Additional file 2: Figure S2**: Boxplots showing the distribution of raw data values for plant height (cm) data under non-stress and drought conditions.

**Additional file 2: Figure S3**: Depiction of the number of same genotypes tested across the years in the breeding program.

**Additional file 2: Figure S4**: Pedigree-based heat map and clustering of the genotypes bred over the years.

**Additional file 2: Figure S5**: Heatmaps showing connectivity of lines across different growing conditions and seasons.

**Additional file 2: Figure S6**: Distribution of the breeding values for grain yield.

**Additional file 2: Figure S7**: Shows genetic trends using a non-parametric approach based on method *loess* under a) under non-stress conditions, b) drought, and c) combined conditions.

**Additional file 2: Figure S8**: Distribution of 200 selected genotypes for grain yield breeding values.

**Additional file 2: Figure S9**: Breeding schemes implemented each year in the drought breeding program from 2003-2019.


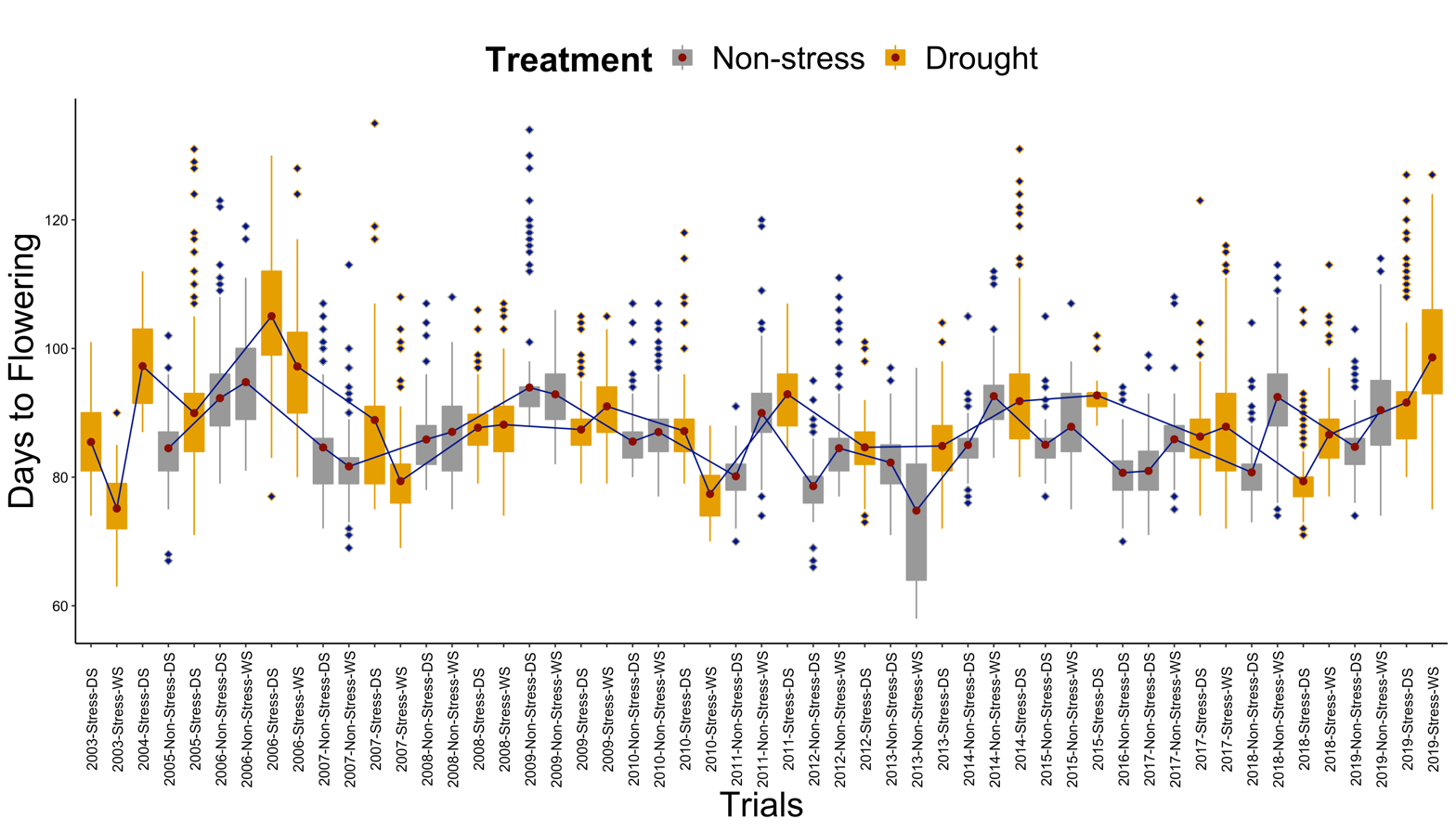


**Figure S1**: Boxplots showing the raw days to 50% flowering (DTF) data under non-stress and drought conditions from 2003-2019. The DTF values of the non-stress trials are illustrated in gray, as a comparison to the DTF values of the drought trials denoted in orange. Outliers are highlighted with blue color.


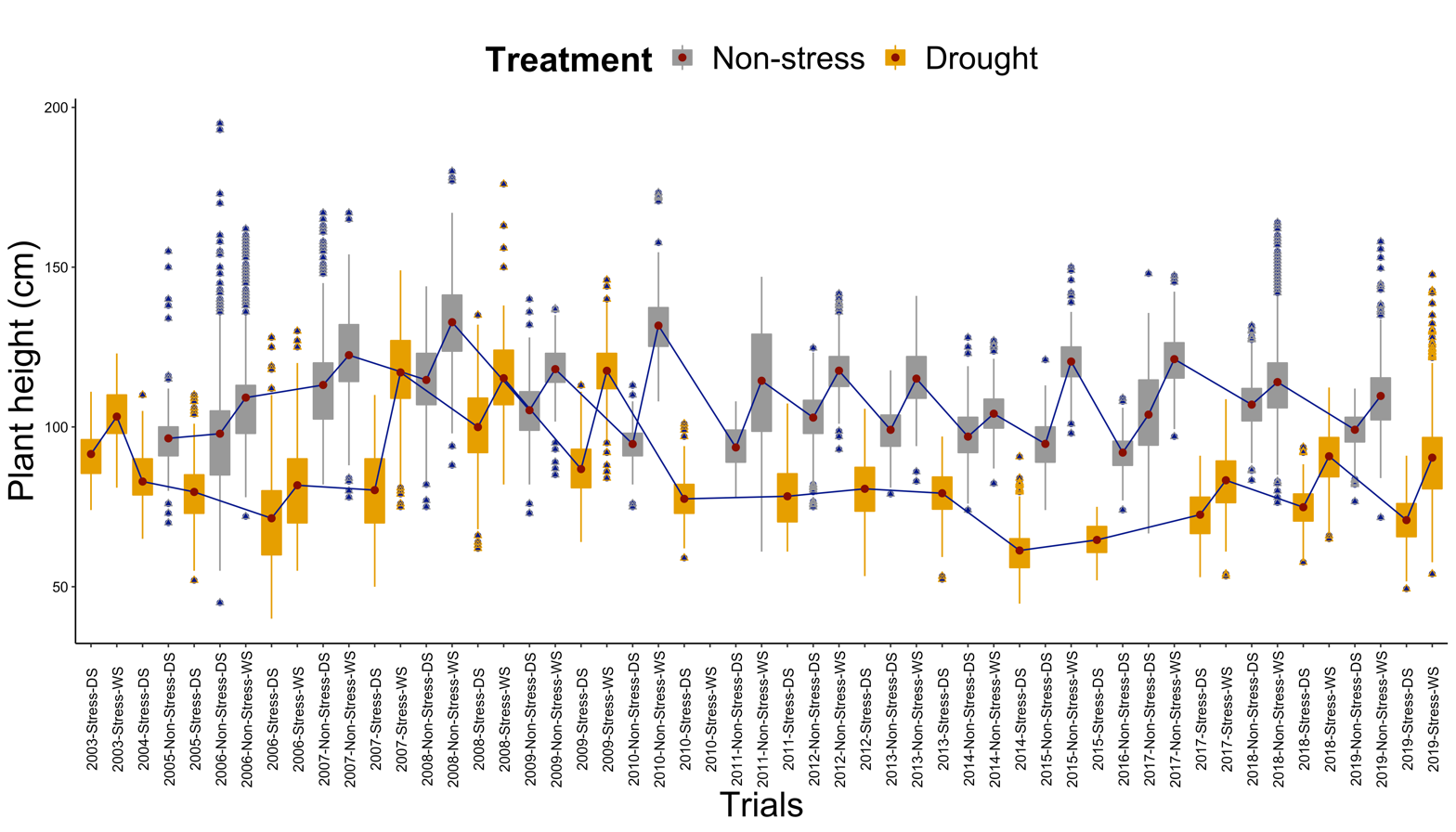


**Figure S2**: Boxplots showing the raw plant height (cm) data under non-stress conditions and drought conditions from the year 2003-2019. The plant height values of the non-stress trials are illustrated in gray, as a comparison to the plant height values of the drought trials denoted in orange.

**
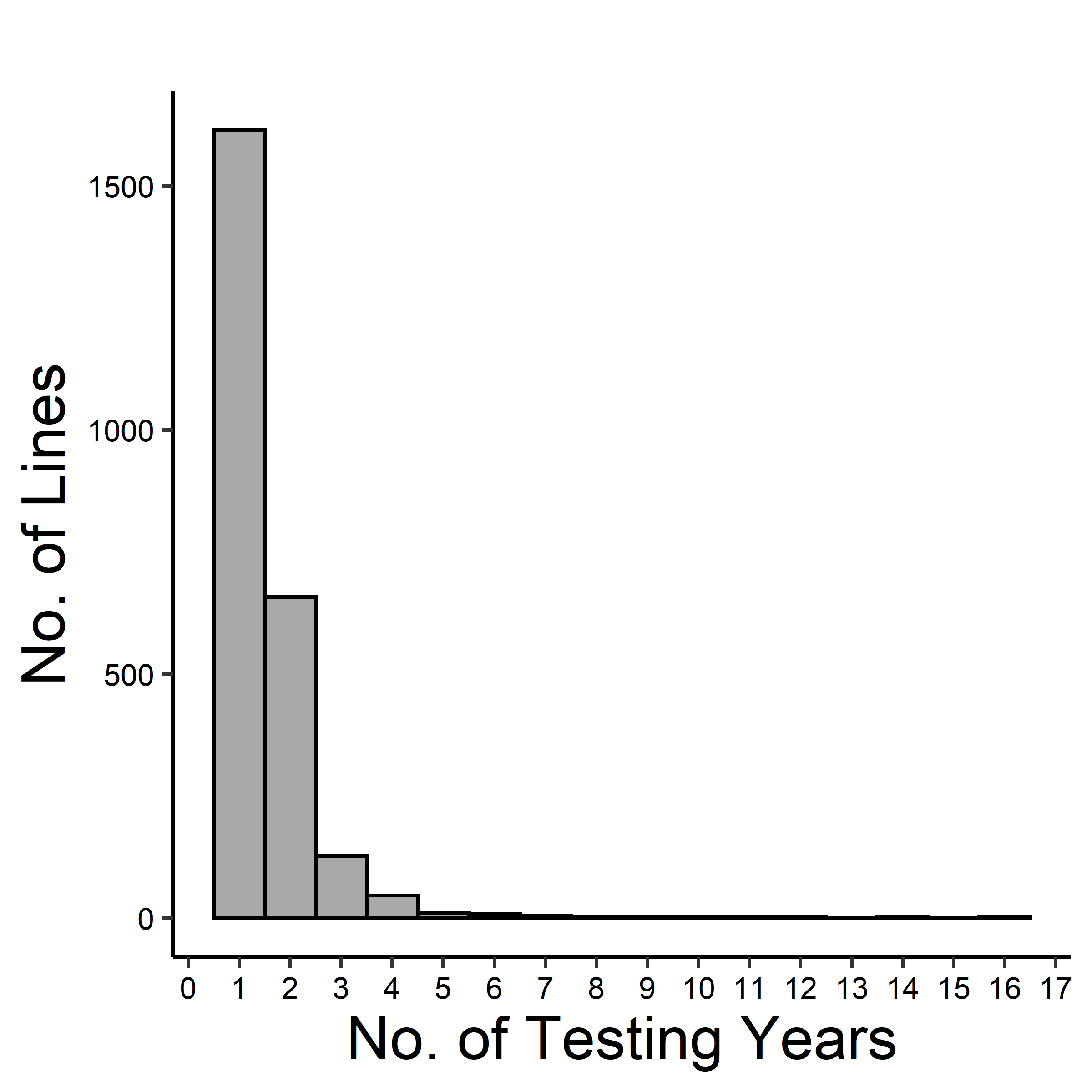
**

**Figure S3**: The figure depicts the number of the same genotypes tested across the years. It is apparent from the figure that a limited number of genotypes were evaluated for more than one year.


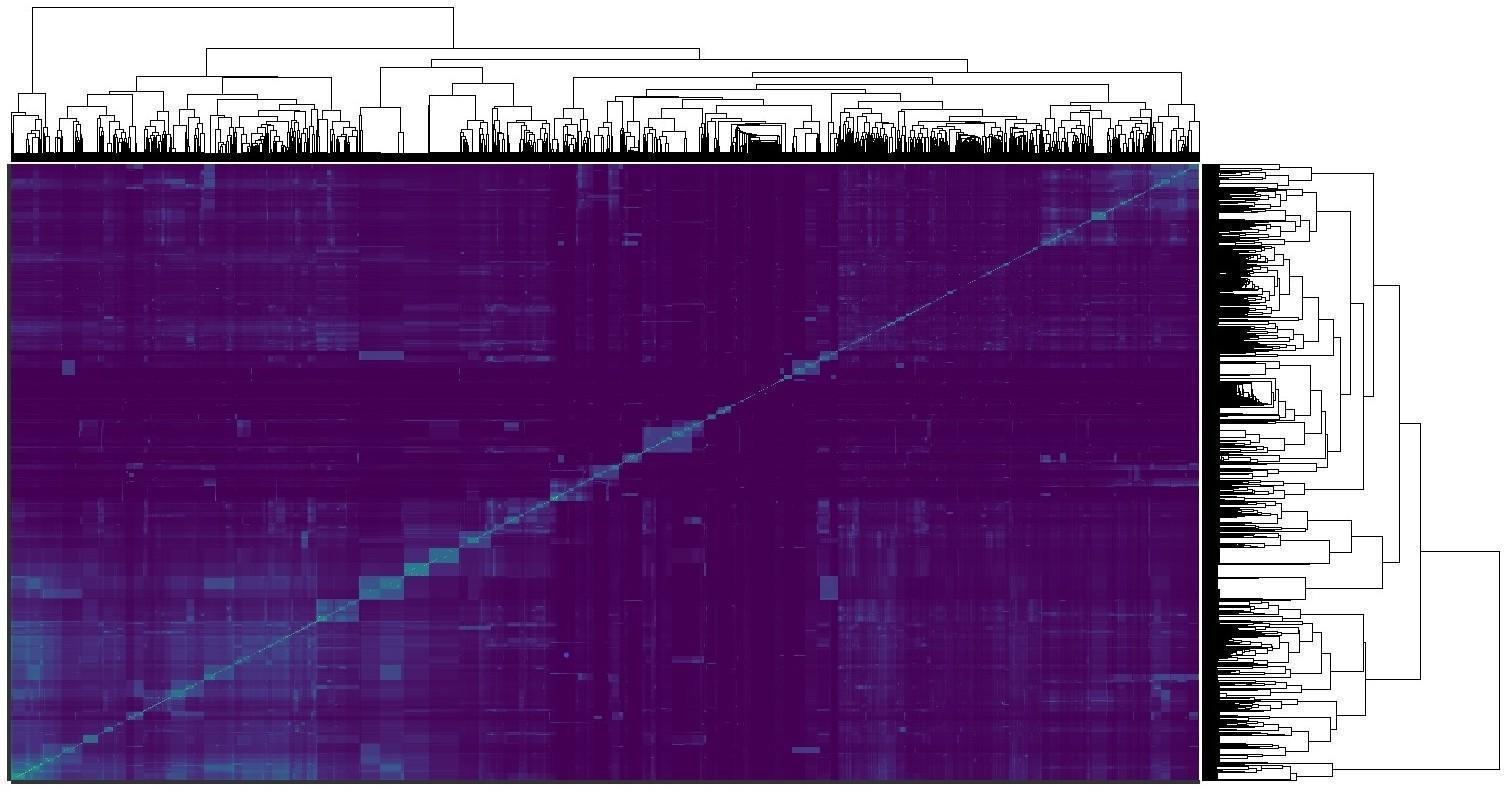


**Figure S4:** Heatmap and grouping of pedigree-based relationship matrix based on 2,490 unique genotypes utilized in IRRI’s rice drought breeding program for 17 years from 2003 to 2019. The figure depicts connectivity between all the lines. The genetically similar lines are depicted in celeste color, which is very well established across the diagonals and evident in the off diagonals indicating the genetic similarity between different genotypes of the breeding program. Each square in the figure represents the sub-group, and in total, 4 main groups were evident, indicating ample diversity in the historical collection.

**Figure S5.** Heatmap depicting the connectivity of the genotypes tested under the (a) non-stress conditions during the dry season, (b) non-stress conditions during wet season, (c) drought conditions during dry season, and (d) drought conditions during wet season. All the heatmaps shows good connectivity tested under different growing conditions.


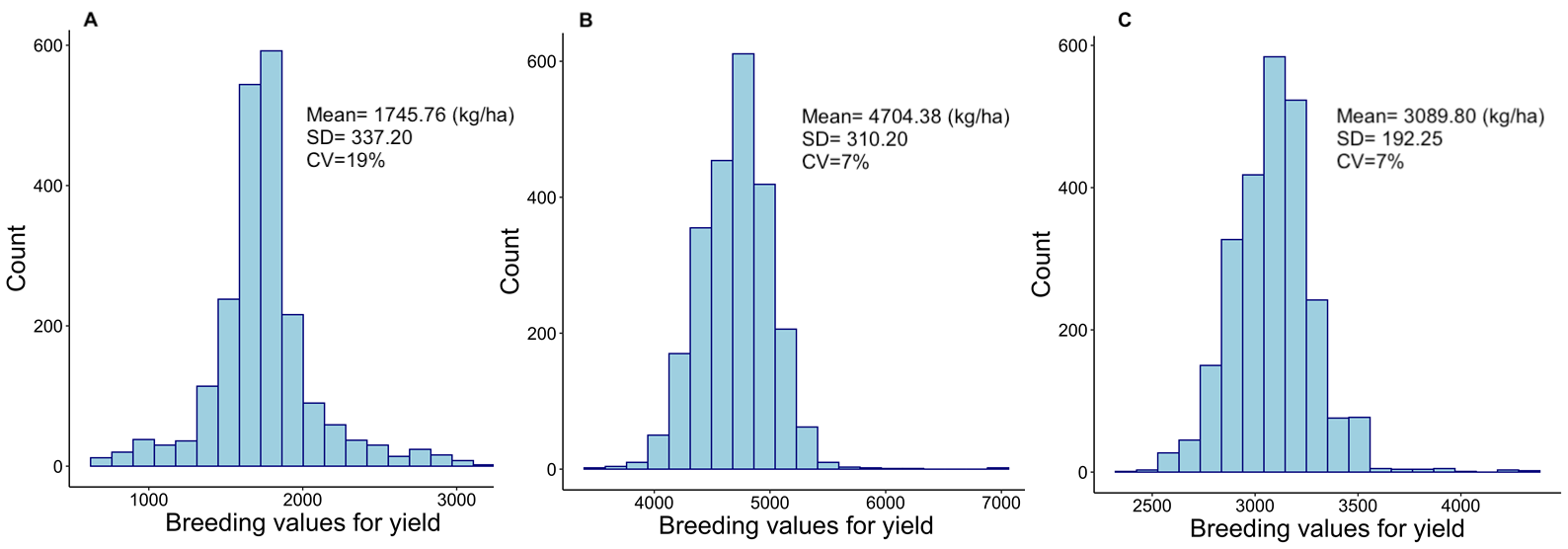


**Figure S6**: Distribution of the breeding values for grain yield (kg/ha) under three conditions: a) Drought, b) Non-Stress, and c). Combined drought and non-stress.


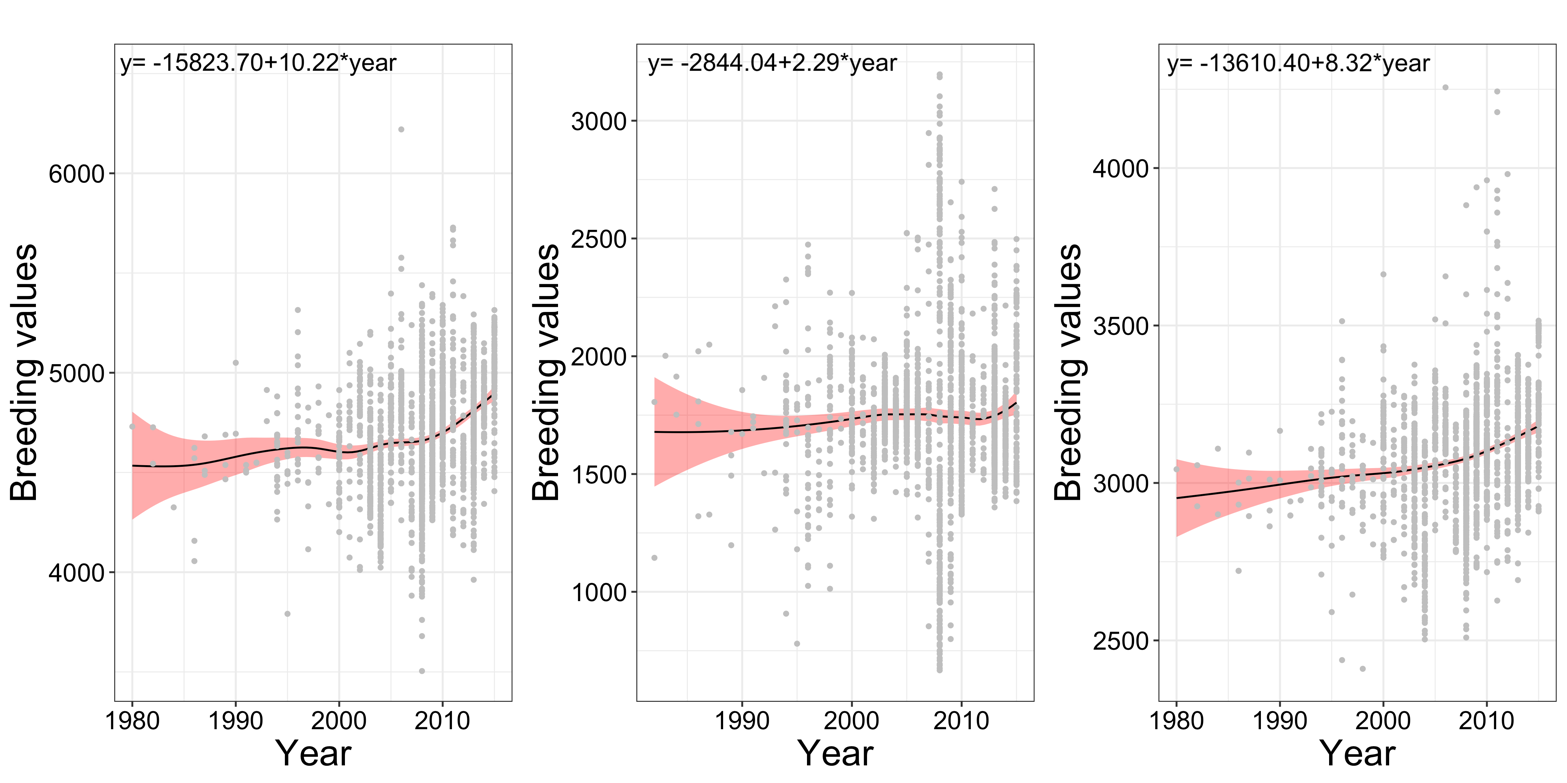


**Figure S7:** Trends in genetic gain from IRRI’s 17 years of drought breeding program under. a) Non-Stress conditions, b) drought conditions, and c) combined conditions (adjusted breeding values under drought and non-stress conditions) using non-parametric approach based on method *loess*. The x-axis shows the year of origin of the genotype ranging from 1980-2015, and the y-axis shows the breeding value of the genotype. The genetic gain was estimated by regressing the breeding values of grain yield on the year of origin of genotype and is given by the slope of the line.

**
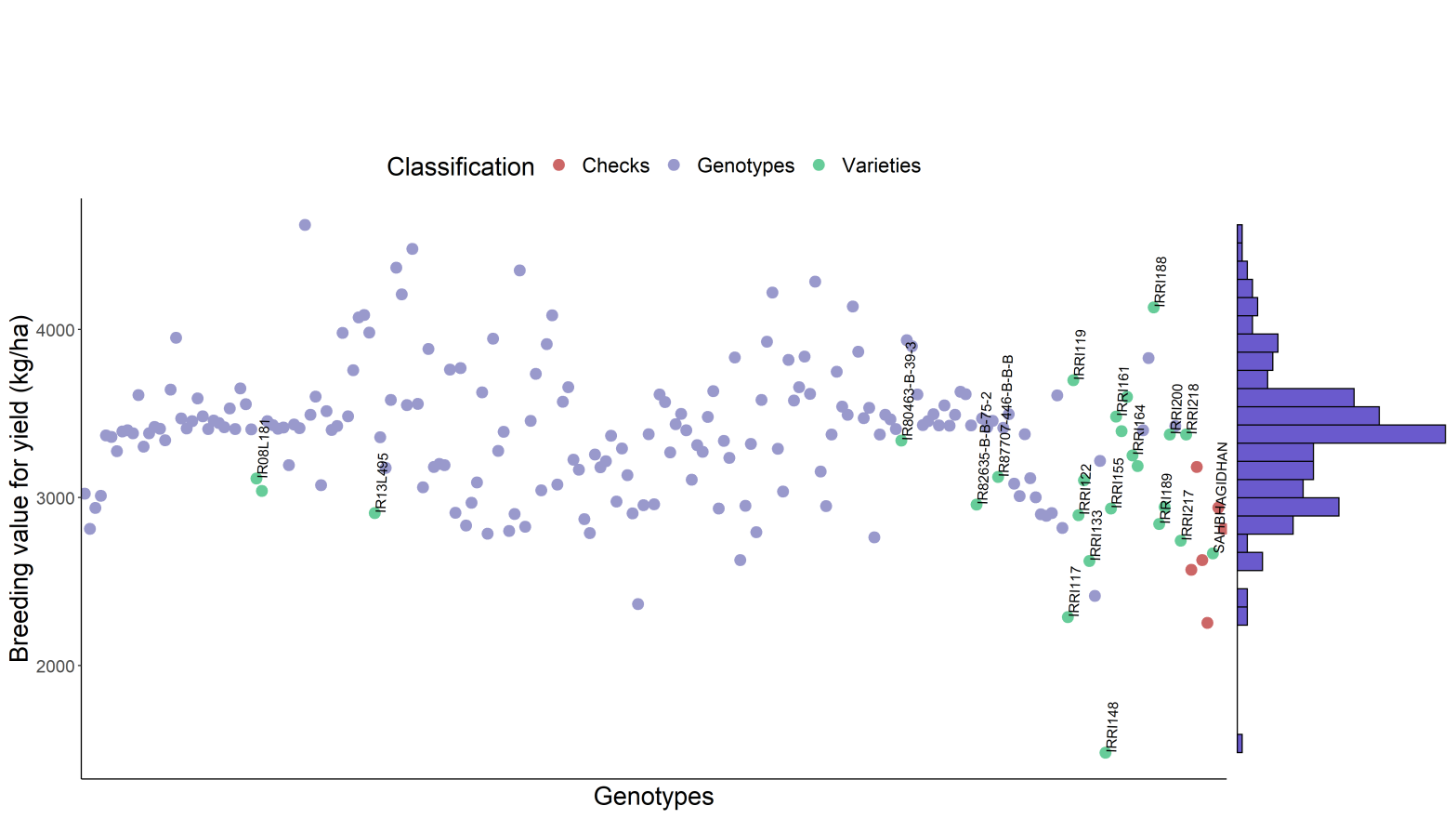
**

**Figure S8:** Representation of the distribution of genotypes comprising the breeding panel. The genotypes include superior genotypes with high breeding values and reliabilities along with additional traits for tolerance to biotic abiotic stresses with superior grain and cooking quality traits. The blue dots represent the selected breeding panel lines which, as depicted by the figure have higher or comparable breeding values compared to the drought-tolerant checks in red and IRRI released varieties in green dots, respectively. The X-axis represents the list of the genotypes, and the Y-axis represents the breeding value for each genotype


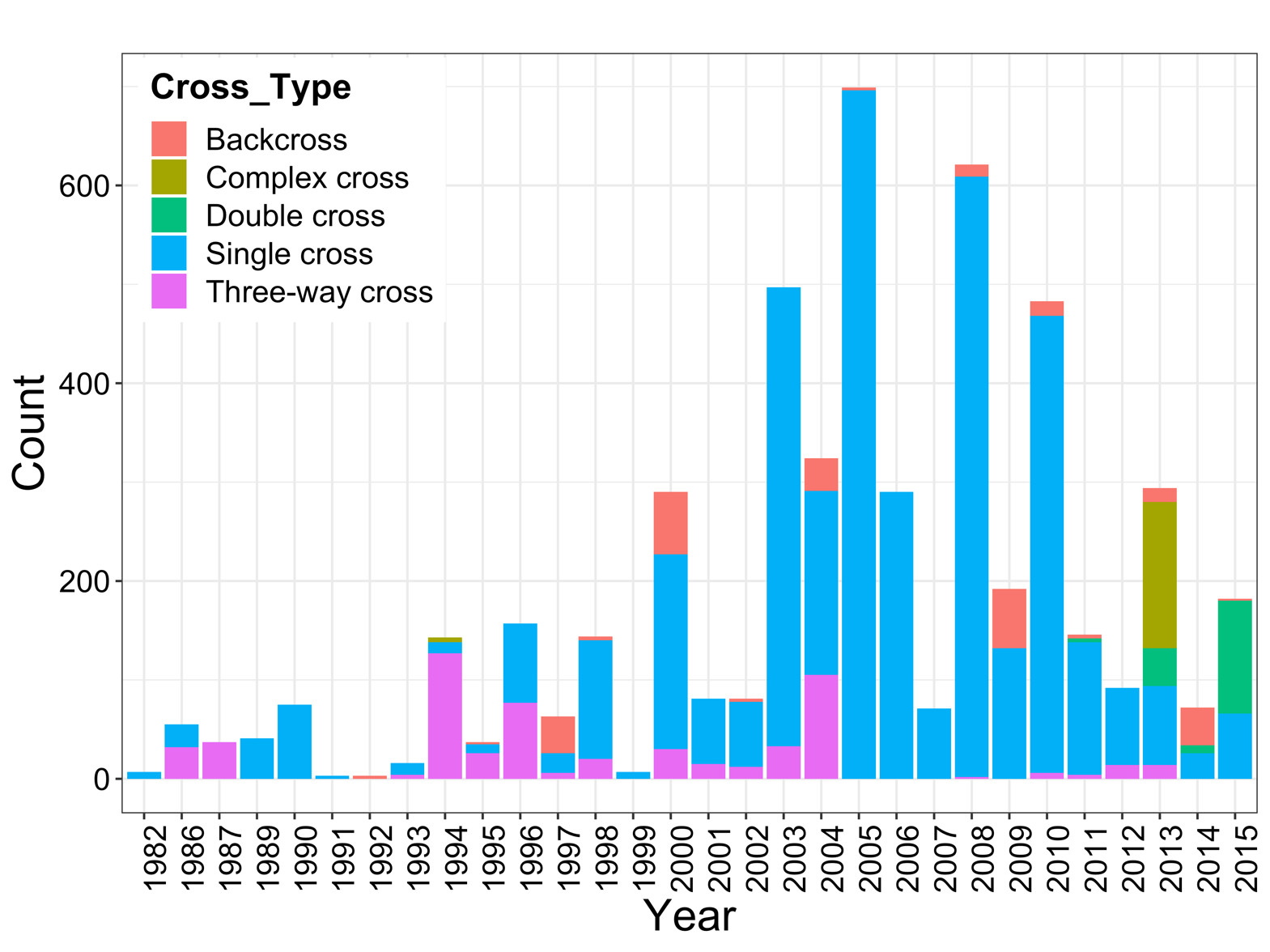


**Figure S9:** The information stating year-wise classification of genotypes developed through various breeding strategies from the year 1980-2015. Mixed types of crossing strategies were adopted by the breeders. In later years, more backcross and complex crosses were made.
